# Effect of Doximity Residency Rankings on Residency Applicants’ Program Choices

**DOI:** 10.5811/westjem.2015.8.27343

**Published:** 2015-11-12

**Authors:** Aimee M. Rolston, Sarah E. Hartley, Sorabh Khandelwal, Jenny G. Christner, Debbie F. Cheng, Rachel M. Caty, Sally A. Santen

**Affiliations:** * University of Michigan Medical School, Ann Arbor, Michigan; † University of Michigan Medical School, Department of Internal Medicine, Ann Arbor, Michigan; ‡ Ohio State University, Department of Emergency Medicine, Columbus, Ohio; § Upstate Medical University, Department of Pediatrics, Syracuse, New York; ¶ Baylor College of Medicine, Houstion, Texas; || University of Michigan, School of Public Health, Ann Arbor, Michigan; # University of Michigan Medical School, Department of Emergency Medicine, Ann Arbor, Michigan

## Abstract

**Introduction:**

Choosing a residency program is a stressful and important decision. Doximity released residency program rankings by specialty in September 2014. This study sought to investigate the impact of those rankings on residency application choices made by fourth year medical students.

**Methods:**

A 12-item survey was administered in October 2014 to fourth year medical students at three schools. Students indicated their specialty, awareness of and perceived accuracy of the rankings, and the rankings’ impact on the programs to which they chose to apply. Descriptive statistics were reported for all students and those applying to Emergency Medicine (EM).

**Results:**

A total of 461 (75.8%) students responded, with 425 applying in one of the 20 Doximity ranked specialties. Of the 425, 247 (58%) were aware of the rankings and 177 looked at them. On a 1–100 scale (100=very accurate), students reported a mean ranking accuracy rating of 56.7 (SD 20.3). Forty-five percent of students who looked at the rankings modified the number of programs to which they applied. The majority added programs. Of the 47 students applying to EM, 18 looked at the rankings and 33% changed their application list with most adding programs.

**Conclusion:**

The Doximity rankings had real effects on students applying to residencies as almost half of students who looked at the rankings modified their program list. Additionally, students found the rankings to be moderately accurate. Graduating students might benefit from emphasis on more objective characterization of programs to assess in light of their own interests and personal/career goals.

## INTRODUCTION

Choosing a residency program is a stressful and important decision for any medical student. The choice of training program will likely influence their future practice and location.^1^ Currently, applicants largely base their decision-making on both 1) personal factors such as geographic location and quality of life; and 2) program factors such as expected clinical experience, curriculum quality, academics, reputation of program, the interview day, and experience with residents and faculty.^2^–^6^ Factors that Emergency Medicine (EM) program directors felt impact applicants’ program choices include the interview experience, personal experience with residents, and academic reputation.^5^

Doximity released residency program rankings by specialty in September 2014 with some collaboration from U.S. News and World Report (USNWR). Doximity is a free, HIPAA-compliant online platform for physicians’ social networking, collaboration, and education. Using their physician network, Doximity administered a survey asking practicing physicians to “nominate up to 5 residency programs in your medical specialty that offer the best clinical training. Do not consider geography. All nominations will receive the same weight regardless of the order in which you list them.”^7^,^8^ More than 17,000 Doximity members responded to the survey, which was conducted between January and July of 2014. Nominations were weighted to account for regional differences in response rates and in the proportion of physicians who are Doximity users.^8^ The result was a ranking of the residencies in each of the 20 surveyed specialties. In addition, Doximity also created the Residency Navigator that includes additional information, when available, such as percentage of graduates from a program who specialize, board pass rate, and alumni with peer review articles, grants or clinical trials. However, this objective information is not included in the ranking lists available to the public as the rankings are “based solely on the reputational component.” Only Doximity members have access to the majority of the added objective data.^9^

The leaders of the national EM organizations responded to the rankings with concern “about the sampling method chosen for this survey, because we believe it will fail to achieve [the] objective for this survey — to identify America’s top EM training programs.”^10^ Arguably, a survey based on reputation alone cannot objectively measure the quality of hands-on-training and other unique aspects of a residency program (e.g., patient acuity, number of procedures, trauma experience, resident satisfaction). The effects of the Doximity findings, which have both reputational and ranking implications, are not yet known, and they could result in changes to applicants’ selections of residency programs. Simply looking at the rank list may bias the candidate when selecting programs for interviews or when ranking programs for the match. Lower ranked programs may suffer the consequences of these rankings by missing quality candidates who may choose not to apply. The objective of this study was to investigate the impact of the Doximity rankings on the program choices made by residency applicants.

## METHODS

### Survey Design

The survey was developed by educational leaders in undergraduate and graduate medical education and senior medical students, all familiar with the residency application process (content validity). The survey was piloted by 20 residents and faculty and revised for response process validity. This study was determined IRB exempt at all three participating schools.

### Survey Content, Administration and Population

The final 12-item survey was sent by email using Qualtrics^TM^ to all fourth year students applying through the National Resident Matching Program at three medical schools in October 2014, just after the release of the Doximity rankings. Student responses were anonymous. Repeated requests were sent by emails weekly to non-responders for three consecutive weeks. The survey initially asked the specialty to which the student applied and whether the student was aware of and looked at the Doximity rankings prior to submitting their application to specific residencies. Students that applied in one of the 20 ranked specialties and who had looked at the rankings were also asked demographic information, how accurate they perceived the rankings on a 100-point scale (0 being not accurate at all and 100 being very accurate), for a narrative to support their score, and whether they added or dropped programs based on the rankings. Additionally, space was provided for students to comment about the rankings.

### Data Analysis

Data analysis included descriptive statistics using SPSS (v22, IBM Corp). Comments were analyzed using grounded theory by a single author. The comments were reviewed, codes identified and then grouped based on common themes. Results were summarized based on these themes.

## RESULTS

A total of 461 students responded to the survey across all three schools (overall response rate of 75.8%), with 425 students applying in one of the 20 ranked specialties by Doximity (see supplemental Table for distribution of specialties). Forty-seven students applied to EM. Of the 425 students applying in one of the ranked specialties, 58% were aware of the rankings and 72% of those aware looked at the rankings (Figure). The demographics of this sample of applicants who looked at the rankings were: mean age 26 years (range 24–33 years), 50% women, 66% self-identified White, 26% Asian and, 5% Black or African American.

Respondents found the rankings moderately accurate with the mean score for accuracy of 56.7 (SD 20.3, range 0–99) for all students; the accuracy rating of the EM applicants was lower with a mean of 43.3 (SD 23.1, range 9–85) (Table 1a and 1b). Of the 114 students who gave justification for their accuracy score, approximately half of them noted that the Doximity rankings did not include all relevant factors in making a choice on a residency program (N=56, 49%). Student comments included “the culture of a program (learning environment, community, resident community) is not reflected in the Doximity scores” and “[it’s] difficult to assess the entire hospital/program based on rankings of different subspecialties. Also difficult to assess patient care vs. research.” On the other hand, 22 (19%) comments indicated that the student felt that the ranking correlated with previous conversations, personal experience, student forums, gut feeling and USNWR rankings. Such comments included “[rankings] appear to align with opinions of advisors” and “There aren’t any other alternatives, and the top programs seemed to align with common knowledge.”

Seventy-nine (45%) of the 177 students who looked at the rankings changed the list of programs to which they applied based on the rankings (Figure). The mean number of programs added and dropped was 4.32 and 2.88, respectively (Table 1a). More specifically, 17% (N=30) both added and dropped programs, 27% (N=47) added programs only, 1% (N= 2) dropped programs only and 49% (N=86) did not add or drop programs, based on the Doximity rankings. Twelve respondents (7%) did not indicate whether they added or dropped programs (Figure). Specifically for students applying to EM, the numbers were similar to the entire sample with a mean number of programs added and dropped of 4.8 and 3.0, respectively.

## DISCUSSION

This study found that medical students utilized and reacted to the Doximity residency rankings with a substantial proportion of participants changing their program choices as a result of viewing the rankings. The majority of students who changed their application list added programs only, increasing the number of programs to which they applied and leading to a potential increased cost. In contrast, some students dropped programs, indicating they excluded residencies initially considered. Lastly, some students were aware of the rankings, and either chose not to check the rankings and/or change their application list.

Students found the rankings, on average, to be only moderately accurate. Our analyses did not break down those that added or dropped programs based on their accuracy rating, but, on average, students appear to be reacting to a ranking they view as only moderately accurate. This may be because students use several pieces of information (all to a varying degree) to make their choices and, therefore, are willing to incorporate an only somewhat reliable source as its impact can be modulated against other pieces of information. Alternatively, this may be a result of the fact that no better data exist.

Some of the accuracy concerns provided by students’ comments highlight potential methodological issues with the Doximity survey that corroborate with the concerns expressed by the leaders of national EM organizations. These potential methodological issues include construct validity which refers to whether an indicator measures what it is intended to measure (i.e., the top programs in the country), and measurement validity which refers to the errors that may ensue in the measurement process.^11^ First, responses were subjective in nature as many physicians do not have first-hand experience with programs other than their own and the ones they attended. Furthermore, there is also a risk of sampling bias with Doximity’s polling methods.^10^ The use of a social media website and inclusion of input from only physicians who are members of Doximity excludes the opinions of many and may sway results based on the characteristics of physicians who sign up for a service such as Doximity.^12^ The rankings are easily manipulated by programs through encouraging their faculty and alumni to join Doximity and cast votes. Lastly, an additional example calling into question the validity of the instrument is that one of the top ten programs in EM was on probation at the time.^13^

Reputation affects decision-making;^14^–^16^ and Doxmity rankings may be a surrogate for reputation for students. However, it is also important to recognize that a number of factors, beyond reputation, influence medical students’ decision-making as they decide which residency programs to which to apply, including objective measurements (such as those included in the Residency Navigator portion of the Doximity report but excluded from the rankings), advising from mentors, and personal reasons. Students also need a means to assess programs and a mechanism to look for specific opportunities that align with their career interests, goals for training, geographical preference, and any influences on family members and personal relationships (such as couples matching). The Doximity rankings could be enhanced by the inclusion of objective data.

Despite substantial research, it is still unclear how we truly make decisions. Emotions and rationality each play a part. For students, the decision to apply to highly ranked programs appeals both to the emotions of success and competition as well as to rationality, which encourages them to choose pathways more likely to lead to success. Other influences on decision-making are biases and heuristics, which are unconscious routines to cope with the complexity inherent in most decision-making.^17^ One heuristic is the assumption that a higher ranked program will provide better training. Additionally, anchoring may lead to weighing certain pieces of information too heavily in the decision-making process. Similarly, confirmation bias leads people to ignore evidence that contradicts their preconceived notions. The rankings can play into these biases and, as a result, students may allow decisions to be based on rankings as a surrogate for quality of training.

Perhaps the best way to aid applicants is to move away from rankings and, instead, provide and focus on objective data about programs that students can judge in light of their own interests, career goals and personal preferences. The concept of providing students a resource such as the Residency Navigator to pull data together might be useful without an overall “ranking.” A process to help programs demonstrate data relevant to finding the right “fit” for a residency and other objective data might include setting (rural vs. urban, public vs. private), academic, research or community focused, board certification scores, in-service training examinations, selectivity, percent of residents progressing on track for specialty milestones, numbers of procedures performed, measurements on the annual ACGME program evaluation, and accreditation and hospital metrics such as Quality Leadership Awards. This information could provide a set of metrics to characterize programs in a transparent fashion. Certain resources serve as a precedent for this.^18^ The Residency Navigator component of the Doximity study attempted to begin such characterization, but it was unfortunately overshadowed by the fanfare of the published rankings. Unless residency programs agree to publish objective data to be used by applicants for best fit, published rankings, such as the one by Doximity, may gain more acceptance and importance over time despite their shortcomings.

## LIMITATIONS

The sample of students applying to EM was small, but they appear similar to the general population in using the Doximity rankings to determine their application list. This small sample size of EM applicants may limit generalizability and future studies should expand the EM sample to other schools. Additionally, surveyed students did not represent all geographic areas, further limiting generalizability. Lastly, recall bias is a potential limitation to these results, as students may not remember exactly how the rankings affected their list. We attempted to limit this effect by surveying students only a few weeks after the initial opportunity for application submission (i.e., September 15).

## CONCLUSION

The Doximity residency rankings by specialty influenced the programs to which fourth year medical students chose to apply. On average, students viewed the rankings as only moderately accurate. These rankings were based on reputation data and did not include objective measures. Rankings are often perceived as offering an objective reality of what is “best.” However, what is best for one applicant may be quite different than what is best for another. Residency applicants would likely be better served by providing students with and focusing them on objective program data that they can consider in terms of their own career and personal goals.

## Figures and Tables

**Figure:**
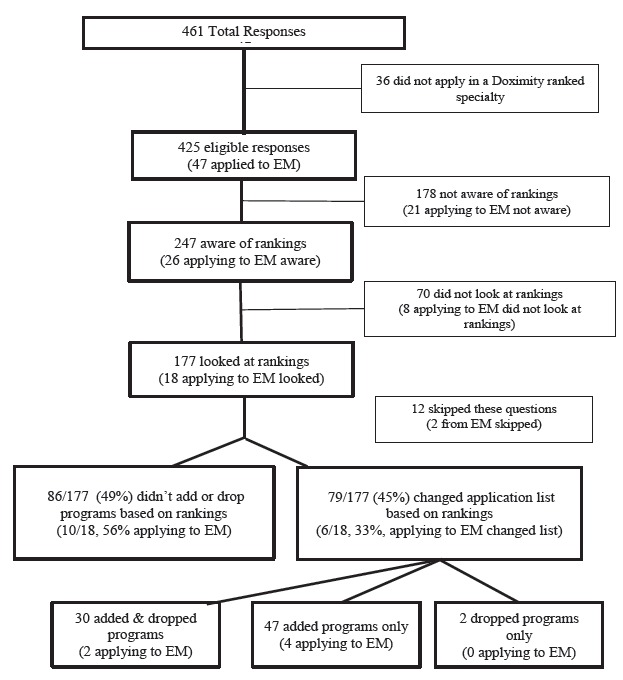
Flow chart showing number of students aware of rankings, that looked at rankings and that modified the list of programs applied to based on rankings.

**Table 1a:** Doximity accuracy ratings and modifications to program list.

	Total sample mean (SD; range)	EM only mean (SD; range)
Accuracy rating (n=162; 17)	56.7 (20.3; 0–99)	43.3 (23.1; 9–85)
Mean number of programs added^a^ (n=77; 6)	4.32 (3.0; 1–11)	4.83 (3.5; 2–11)
Respondents dropping programs^a^ (n=32; 2)	2.88 (2.2; 1–11)	3.00 (1.4; 2–4)

EM, emergency medicine

a Scale of “1” – “>10”. Note that choice of “>10” programs added or dropped was coded as “11” for purposes of determining the mean.

**Table 1b:** Factors and reasoning for modifications.

	Total sample number (%)	EM only number (%)
Reasoning for adding or dropping		
Added programs highly ranked	62 (39%)	4 (25%)
Dropped programs lowly ranked	20 (13%)	0 (0%)
Added “safety” programs	25 (16%)	4 (25%)
Added “reach” programs	24 (15%)	2 (13%)
Other	2 (1%)	0 (0%)
Important factors		
Couples matching	24 (15%)	5 (28%)
Geographic location	150 (91%)	17 (94%)
Academic Reputation	146 (89%)	15 (83%)
Personal Connection	99 (60%)	13 (72%)
Choice to stay at home institution	37 (22%)	5 (28%)
Other	18 (11%)	0 (0%)
